# Neural Style Transfer as Data Augmentation for Improving COVID-19 Diagnosis Classification

**DOI:** 10.1007/s42979-021-00795-2

**Published:** 2021-08-13

**Authors:** Netzahualcoyotl Hernandez-Cruz, David Cato, Jesus Favela

**Affiliations:** 1 Ulster University, Belfast, UK; 2 Independent Researcher, London, England, UK; 3 CICESE, Ensenada, Baja California Mexico

**Keywords:** Generative adversarial network, Transfer learning, Convolutional neural network, Neural style transfer, Data augmentation

## Abstract

Coronavirus disease 2019 (COVID-19) has accounted for millions of causalities. While it affects not only individuals but also our collective healthcare and economic systems, testing is insufficient and costly hampering efforts to deal with the pandemic. Chest X-rays are routine radiographic imaging tests that are used for the diagnosis of respiratory conditions such as pneumonia and COVID-19. Convolutional neural networks have shown promise to be effective at classifying X-rays for assisting diagnosis of conditions; however, achieving robust performance demanded in most modern medical applications typically requires a large number of samples. While there exist datasets containing thousands of X-ray images of patients with healthy and pneumonia diagnoses, because COVID-19 is such a recent phenomenon, there are relatively few confirmed COVID-19 positive chest X-rays openly available to the research community. In this paper, we demonstrate the effectiveness of cycle-generative adversarial network, commonly used for neural style transfer, as a way to augment COVID-19 negative X-ray images to look like COVID-19 positive images for increasing the number of COVID-19 positive training samples. The statistical results show an increase in the mean macro *f*1-score over 21% on a one-tailed *t* score = 2.68 and *p* value = 0.01 to accept our alternative hypothesis for an $\alpha = 0.05$. We conclude that this approach, when used in conjunction with standard transfer learning techniques, is effective at improving the performance of COVID-19 classifiers for a variety of common convolutional neural networks.

## Introduction

Coronaviruses are a family of microorganisms known to cause respiratory infections. The most recent outbreak Coronavirus,Disease 2019 (COVID-19) [5] is spreading worldwide [2, 31]. Symptoms of COVID-19 share some visual similarities with other common respiratory diseases such as pneumonia [20] when observed using an X-ray scanning machine. One of the routine test techniques currently used to assist in diagnosing COVID-19 consists of chest radiological imaging such as computed tomography and X-ray radiographs [38]. Chan et al. [3] show evidence that early symptoms can be observed in X-rays in infected areas of a patient’s chest. Similarly, Yoon et al. [35] have shown that X-ray images contain features helpful in distinguishing COVID-19 from other respiratory diseases, such as opacity in the lower lung, hence providing a more accurate diagnosis of COVID-19.

In the context of image classification problems, convolutional neural networks (CNNs) can be effective at supervised image-based classification tasks. Yet, they typically entail a large amount of labelled training data to be effective [11]. Some early thriving CNNs such as AlexNet [13] and GoogLeNet [29] require training with thousands of labelled samples per class [28], which presents a challenge when applying these solutions to emerging problem areas, such as the diagnosis of the ongoing COVID-19 pandemic, where the availability of labelled data is limited.

Two techniques increasingly being used to improve the performance of a classifier with a small number of labelled samples are transfer learning and data augmentation. Transfer learning is a technique in which a model is first trained on a general task for which there exists a large training dataset and then fine-tuned on a related specific task [9]. When successful, good performance can be achieved on the fine-tuning task using relatively few additional data samples. Data augmentation for image classification is a strategy that increases the diversity of existing training data by performing geometric transformations, colour space augmentations, feature space augmentation, adversarial training, and generative adversarial networks (GANs) [26]. The benefit of using GANs is that they enable transforming data samples from one class into another class using techniques created for neural style transfer [6].

In this paper, we illustrate the effectiveness of data augmentation by applying the neural style transfer approach to alleviate the insufficiency of labelled samples for image classification problems in unbalanced datasets. We rely on generating synthetic images from the under-represented class. Specifically, we use GANs to synthesise COVID-positive X-ray images from publicly available X-ray images of healthy patients and patients diagnosed with pneumonia, which given its visual similarity to COVID-19, challenging the capabilities of CNNs classifiers. Viewing this task as a multi-class problem with three classes: X-ray images from healthy patients, diagnosed with pneumonia, and diagnosed with COVID-19, this paper is driven by the following research question and hypothesis:

*Q*: To what extent can the use of synthetic images improve CNN’s ability to classify X-ray images?

From this, we derive the following hypothesis:

$$H_{1}$$:
Synthetic images improve the performance of CNNs in multi-class classification problem to accurately distinguish X-ray radiographs from healthy patients, patients diagnosed with respiratory diseases such as pneumonia, and patients diagnosed positive for COVID-19.

The paper begins by introducing related work in Section “Related Work”, followed by a description of our approach to generate COVID-19 positive samples in Section “Feature Transfer Using a Cycle-Generative Adversarial Network”. We outline our experiments in Section “Experimental Investigation” and present our results in Section “Results”. We conclude with Section “Discussion and Conclusion”, where we discuss our results and propose further directions.

## Related Work

### Synthetic Images Using Generative Adversarial Networks

GANs are a type of machine learning model consisting of two neural networks, a Generator and a Discriminator. While the Discriminator classifies data into two classes—realistic or unrealistic, the Generator produces fake data and is rewarded for fooling the Discriminator [6]. GAN’s training is successful when the Generator learns to generate data that is indistinguishable from the training set [7].

Several architectures for GANs have been proposed. For instance, Super Resolution GANs (SRGAN) consists of three neural networks: a very deep generator network, a discriminator network, and a pre-trained VGG-16 network. It uses a perceptual loss function defined as the weighted sum of the content loss and adversarial loss to achieve a natural and more sufficient detail [16], which is commonly used when high-resolution images are available. InfoGAN (Information GANs) uses concepts from information theory such that the noise term is transformed into latent codes which provide predictable and systematic control over the output. It maximises the mutual information between a small subset of the latent variables and a given observation sample, where labels act as an extension to the latent space to generate and discriminate images better [4], which makes it inconvenient for scenarios of scarce data. Cycle-Generative Adversarial Network (CycleGAN), however, adapt significant features from a given domain to an image from another domain. It learns to perform image translation in the absence of training pairs [37], which makes it convenient for scenarios with insufficient data.

### Implementation of Transfer Learning Technique for Convolutional Neural Network Architectures

CNNs are multi-layer neural networks designed to recognise visual patterns. In supervised image classification, two key factors in the success of CNNs are the availability of labelled data and the topology of CNNs’ architecture.

The availability of labelled data has a strong influence on the performance of a CNN as it can affect the quality of the features to characterise the different categories included in a given dataset. The CNN architecture contributes an inductive bias for spatial invariance, which helps identify the most appropriate features characteristic of an image category. Efforts aligned to the detection of COVID-19 include the adaptations of CNNs such as DarkNet (DarkCovidNet [20]), DenseNet (CovidAID [18]), VGG-19 (VGG-19 [2]), and ResNet (ResNet50+SVM [24]) architectures, which report classification performances of 87%, 92%, 93%, and 95% for a macro *f*1-score; respectively. DarkNet, for example, is an architecture that introduces a filter layer in each of the convolutional layers of the architecture, ResNet50+SVM extracts features from the fully connected CNN which are fed into an SVM for classification. CovidAID and VGG-19 vary in the classification layers from the respective CNNs architectures. These architectures leverage the feature mining capabilities of transfer learning to improve the convolutional modelling, a downside, however, is that they require above 1000 samples of X-ray images for training purposes, which presents a challenge when access to data is limited.

CNN algorithms became fundamental in modern architectures. Its importance goes back to 2012 when modern CNN introduced the concept of depth in neural networks [15] and showcased their impact at the ImageNet Large Scale Visual Recognition Challenge (ILSVRC) [22] by outperforming other alternatives by a difference of 10.7%.

To date, some of the most successful architectures consist of deep structures demanding sizeable computational power to address complex architectures comprised of millions of parameters and large datasets assembled by millions of images. Nevertheless, in practice, having access to both computational power and a sufficient amount of labelled data is a challenge. Modern strategies such as transfer learning [34] enable an approach wherein a pre-trained CNN retains both its initial architecture and the learned experience (e.g. weights). In this context, using transfer learning, one can make use of pre-trained models (computed over million of samples) to predict new categories alleviating the need for a large dataset and computational resources.

Transfer learning techniques have proved useful for classification problems hampered by the insufficiency of labelled data. Previous research has shown that with as little as 50 samples, one can fine-tune a pre-trained model to achieve a classification accuracy above 90% [20, 25, 32].

In what follows, we briefly describe some of the most relevant CNN architectures discussed in this work.

- *AlexNet* Architecture winner of ILSVRC 2012 was proposed by Krizhevsky et al. [14]. It consists of fixed kernels of size 11$\times$11, 5$\times$5, and 3$\times$3. It comprises five convolutional layers, some of which are followed by max-pooling layers, and two globally connected layers with a final 1000-way softmax. It attached ReLU activation function after every convolutional and fully-connected layer. To be used for transfer learning, the 6th layer of the classifier needs to be retrained.
- *DenseNet* Architecture was proposed by Huang et al. [10]. It connects each layer to every other layer in a feed-forward fashion. Whereas traditional convolutional networks with L layers have L connections, one between each layer and its subsequent layer, DenseNet has *L*(*L* + 1)/2 direct connections. To be used for transfer learning, the last fully connected linear layer needs to be retrained.
- *ResNet* Architecture was winner of ILSVRc 2015 proposed by He et al. [8]. It consists of a network of 152 layers. It introduced a novel architecture with skip connections and features heavy batch normalisation connections also known as gated units. To be used for transfer learning, the last fully connected linear layer needs to be retrained.
- *ResNeXt* Architecture was proposed by Xie et al. [33]. It is a highly modularised network constructed by repeating a building block that aggregates a set of transformations with the same topology. To be used for transfer learning, the last fully connected linear layer needs to be retrained.
- *SqueezeNet* Architecture was proposed by Landola et al. [12]. It begins with a standalone convolution layer, followed by eight fire modules, ending with a final convolutional layer. Where a fire module comprised a squeeze convolution layer (which has only 1$\times$1 filter), feeding into an expand layer that has a mix of 1$\times$1 and 3$\times$3 convolution filters. To be used for transfer learning, the last fully connected linear layer needs to be retrained.
- *VGG* Architecture was proposed by Oxford university’s Visual Geometry Group [27]. It consists of 16 convolutional layers thought a uniform fully connected architecture. To be used for transfer learning, the 6th layer of the classifier needs to be retrained.

Other CNNs referenced in this work are GoogLeNet [29], MNASNet [30], ShuffleNet [17], Wide ResNet [36].

## Feature Transfer Using a Cycle-Generative Adversarial Network

In this paper, we adopted CycleGAN as a technique to generate augmented X-ray radiographs of patients diagnosed as positive for COVID-19 (hereafter referred to as *covid-synthetic* ${\hat{Y}}$). As described before, this technique’s foundation relies on two CNNs which iterate to transform an image from a source category *X* so that it looks as if it belongs to a target category *Y* [37].

### Discriminator Component

The discriminator component consists of a CNN that sees an image and classifies it as realistic or unrealistic.

**Fig. 1:**
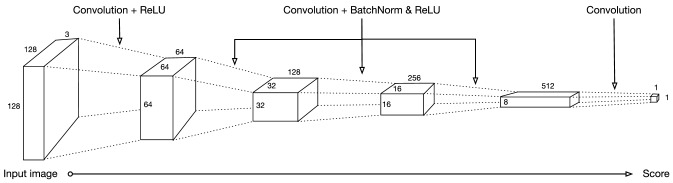
Architecture for the CycleGAN’s Discriminator CNN

As presented in Fig. 1, the Discriminator sees a three dimensional image of size 128$\times$128$\times$3 (width, height, depth, respectively) and passes it over five convolutional layers with a down-sampling factor of two. The first four layers apply a BatchNorm and ReLu activation function, while the last layer acts as a classifier which outputs a scalar value. The output is normalized between 0 and 1 and treated as a probability score where realistic *covid-synthetic* images are close to 1, and unrealistic *covid-synthetic* images are close to 0.

### Generator Component

**Fig. 2:**
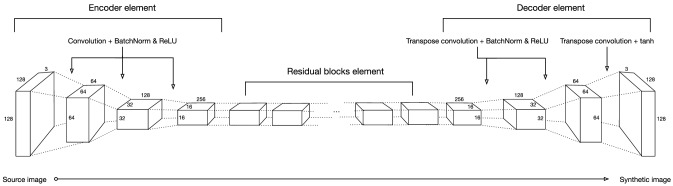
Architecture for the CycleGAN’s Generator CNN

Figure 2 shows the generator component, which consists of three elements. The first two elements define a convolutional encoder network as feature extractor and a convolutional decoder network as a feature assembler, whereas a third element establishes a network of residual block [8].

The encoder network sees a 128$\times$128$\times$3 image and down-samples it by a factor of two resulting in a compressed 16x16x256 tensor. The compressed image is then passed through a network of residual blocks, followed by the decoder network that up-samples the output of the residual block, which outputs a synthetic image.

Note that while the ReLU activation function is used for the convolutional layers, the final transposed convolutional layer uses tanh as activation function since it has been reported to be appropriate at addressing saturation and colour symmetry in images [21].

**Fig. 3:**
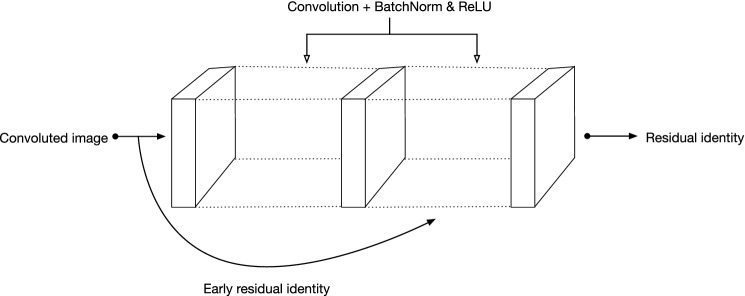
Representation of a single residual block

The residual block consists of six CNNs with the same size input and output, where each network is built upon two convolutional layers using ReLU activation function on the output of the first layer and BatchNorm to the outputs of both layers as depicted in Fig. 3.

### Training Pipeline

**Fig. 4:**
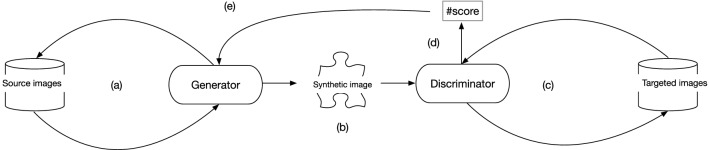
Workflow for generating synthetic images over a CycleGAN

The workflow for generating synthetic images consists of a sequence of iterative steps driven by the pipeline of the CycleGAN. As illustrated in Fig. 4, the process starts when the Generator is given category of images *X* as input (a) and generates a synthetic image (b). The Discriminator then analyses the images from the targeted category *Y* (c) and returns a score estimating the similarity of the synthetic output to a realistic or unrealistic appearance (d). The Generator uses this score to update its weights using back-propagation (e), followed by the reconstruction of the synthetic image.

Every step in this pipeline is repeated for a given number of iterations (*n*) to continuously improve the performance of the network.

## Experimental Investigation

The approach was tested using a Tesla NV100^1^ data centre consisting of eight GPUs. We designed two experiments in which we used a publicly available unbalanced dataset of X-ray images collected from 43 publications [19].

### Unbalanced Dataset

The dataset consisted of 2905 labelled X-ray samples classified in one of the following three categories: patients confirmed as having no relevant medical diagnoses (*healthy*, *n* = 1341), patients having a confirmed pneumonia diagnosis (*pneumonia*, *n* = 1343), and patients having a confirmed COVID-19 diagnosis (*covid*, *n* = 219). The sample data consisted of conventional radiographic images represented as a grayscale image with resolutions ranging from 477$\times$411 to 2169$\times$1852 pixels (width $\times$ height).

### Unpaired Source-Target Sets to Train the CycleGAN

We first trained the CycleGAN to learn to capture the distinctive characteristics of one category to transfer them into another category in the absence of any paired training examples. In this context, we used the *healthy* category as the source set *X* and the *covid* category as the target set *Y*. The model outputs images resembling those from the category *Y*, so we refer to outputs belonging to this category as *covid-synthetic* ${\hat{Y}}$.

**Fig. 5:**
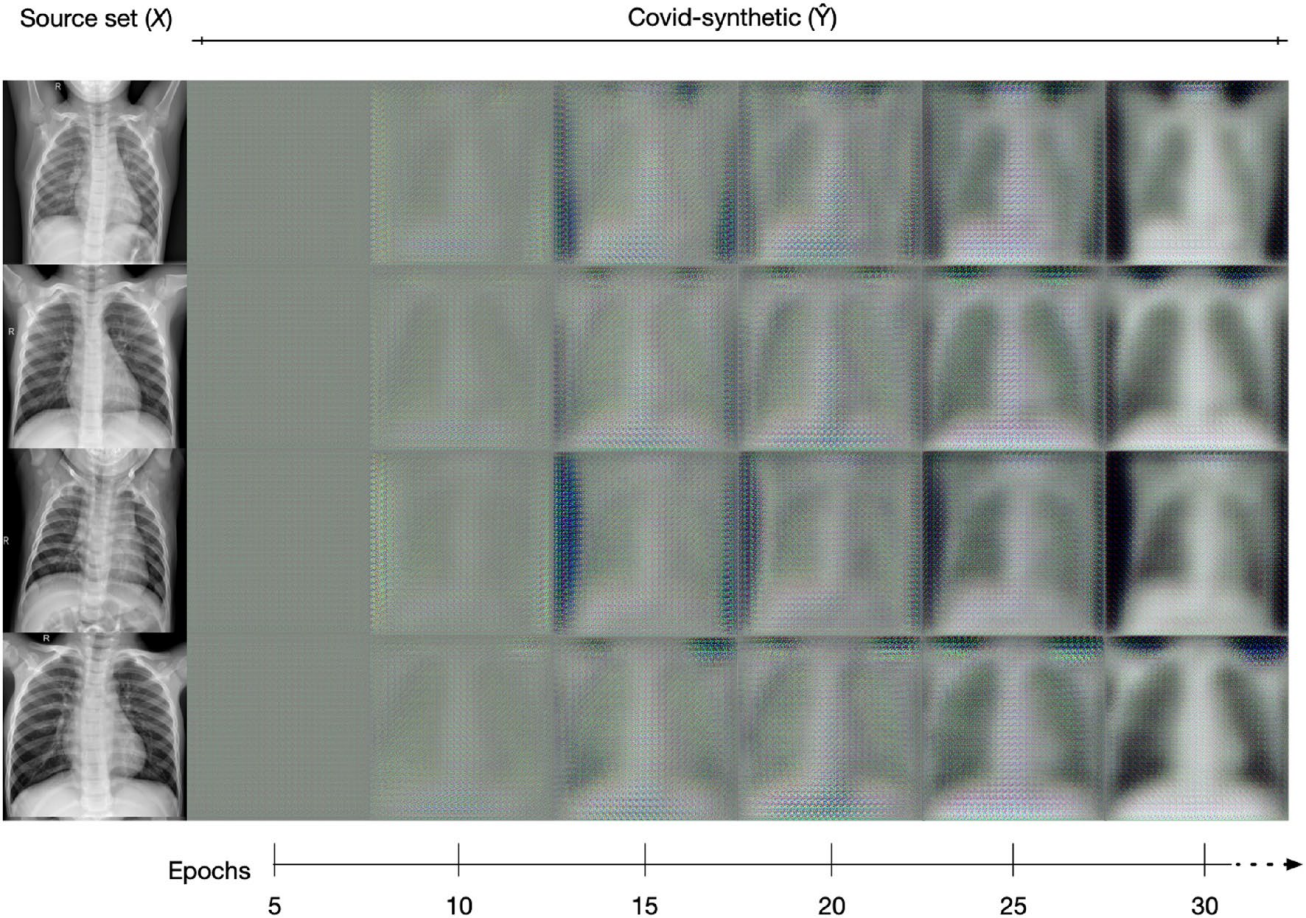
Sample of images to illustrate the creating of *covid-synthetic* images derived from images of the *healthy* category. It can be observed that the quality of synthetic images improves as the training progresses. The leftmost column shows images from the *healthy* category and the remaining columns show the progression of the *covid-synthetic* image generation process over 30 epochs

To populate the *covid-synthetic* category, we trained the CycleGAN under an unsupervised approach in which the source and target image sets are unpaired, *i.e.* $X \ne Y$. The source set *X* consists of 1341 *healthy* images, whereas the target set *Y* consists of 219 *covid* images. This approach aims to provide sufficient data for the CycleGAN to capture the distinctive characteristics from *Y* and transfer them to images from *X*. Note that as this approach eliminates the need for source-to-target image pairs, it leverages the scarce amount of images in *Y* by transferring key features to the widely populated *X*. For the purposes of this work, we generate ${\hat{Y}}$ consisting of 100 *covid-synthetic* images (Fig. 5).

To train the CycleGAN, we used Adam optimiser and defined the model’s hyperparameters using a learning rate of 0.0001 and beta values: 0.5, 0.999. The training images were resized to fit 224$\times$224$\times$3 tensors [21]. As a preprocessing step, we scaled the images from –  1 to 1-pixel range; as it has been reported appropriate to address image saturation [21]. The CycleGAN was trained for ten thousand epochs ($\approx$ 72 h).

### Fine-Tuning Pretrained CNNs with Transfer Learning

The second experiment aims at balancing a dataset by incorporating images from the *covid-synthetic* category. We evaluate the benefit of using synthetic images by analysing their contribution in the performance of different CNNs. Thus, here we present our baseline and six alternatives to training the CNNs by gradually incorporating images from the *covid-synthetic* category.

To evaluate the benefit of adding *covid-synthetic* images to train supervised machine learning algorithms, we adapted ten common CNNs (Section “Implementation of Transfer Learning Technique for Convolutional Neural Network Architectures”) using transfer learning technique. Hence, we fine-tune by freezing the pre-trained weights up to, but not including, the last layer and only retrain the weights from the last convolutional layer.

As preparation, we ensured that the input images were preprocessed consistent with the original method used to train the CNNs, including resizing and normalisating the inputs (mean = [0.485, 0.456, 0.406] and std = [0.229, 0.224, 0.225]). We used the Adam optimiser with learning rate: 0.0002 and beta values: 0.5, 0.999 as hyperparameters [14].

The CNNs were trained for 50 epochs, but only the model with the best validation score was considered.

Driven by our research question “To what extent can the use of synthetic images improve CNNs ability to classify X-ray images?”, we trained the CNN models with six different datasets assembled over *covid-synthetic* images and compared their performance by looking at the macro *f*1-score.

The baseline CNN was trained over 15 images across the three categories (*healthy*, *pneumonia*, and *covid + covid-synthetic*), whereas the six CNN models were trained over a balanced distribution of images in which synthetic images were gradually including as presented in Table 1.

**Table 1:** Distribution of labelled samples across the assembled training datasets

	Healthy	Pneumonia	Covid + *covid-synthetic*
Baseline	15	15	15 + **0**
(+) 15 GAN	30	30	15 + **15**
(+) 30 GAN	45	45	15 + **30**
(+) 45 GAN	60	60	15 + **45**
(+) 60 GAN	75	75	15 + **60**
(+) 75 GAN	90	90	15 + **75**
(+) 90 GAN	105	105	15 + **90**

The first setting defines the baseline, whereas the rest represent six variations in which *covid-synthetic* samples are gradually included (left column)

For validation purposes, we used a subset of unseen samples consisting of 30 images from each of the dataset’s categories (*healthy*, *pneumonia*, and *covid*), and 174 for the testing stage where $X \ne Y$ (Table 2). Note that images from the augmented category *covid-synthetic* are used to enhance the accuracy of our CNN models; hence they are not included as part of the validation dataset.

**Table 2:** Distribution of labelled X-ray radiographs

Category	Validate	Test	Total
Healthy	30	174	**204**
Pneumonia	30	174	**204**
Covid	30	174	**204**
Total	**90**	**522**	**612**

## Results

Here we show the results in three sections. We first analyse output samples of synthetic images. Next, we discuss the transfer learning performance over ten of the most common open-access CNNs across six different models trained over *covid-synthetic* images. Finally, we present a theoretical evaluation through a hypothesis test using one-tailed *t* score and *p* values for $\alpha =0.05$.

### Comparison Between Source Samples and CycleGAN Generated Samples

**Fig. 6:**
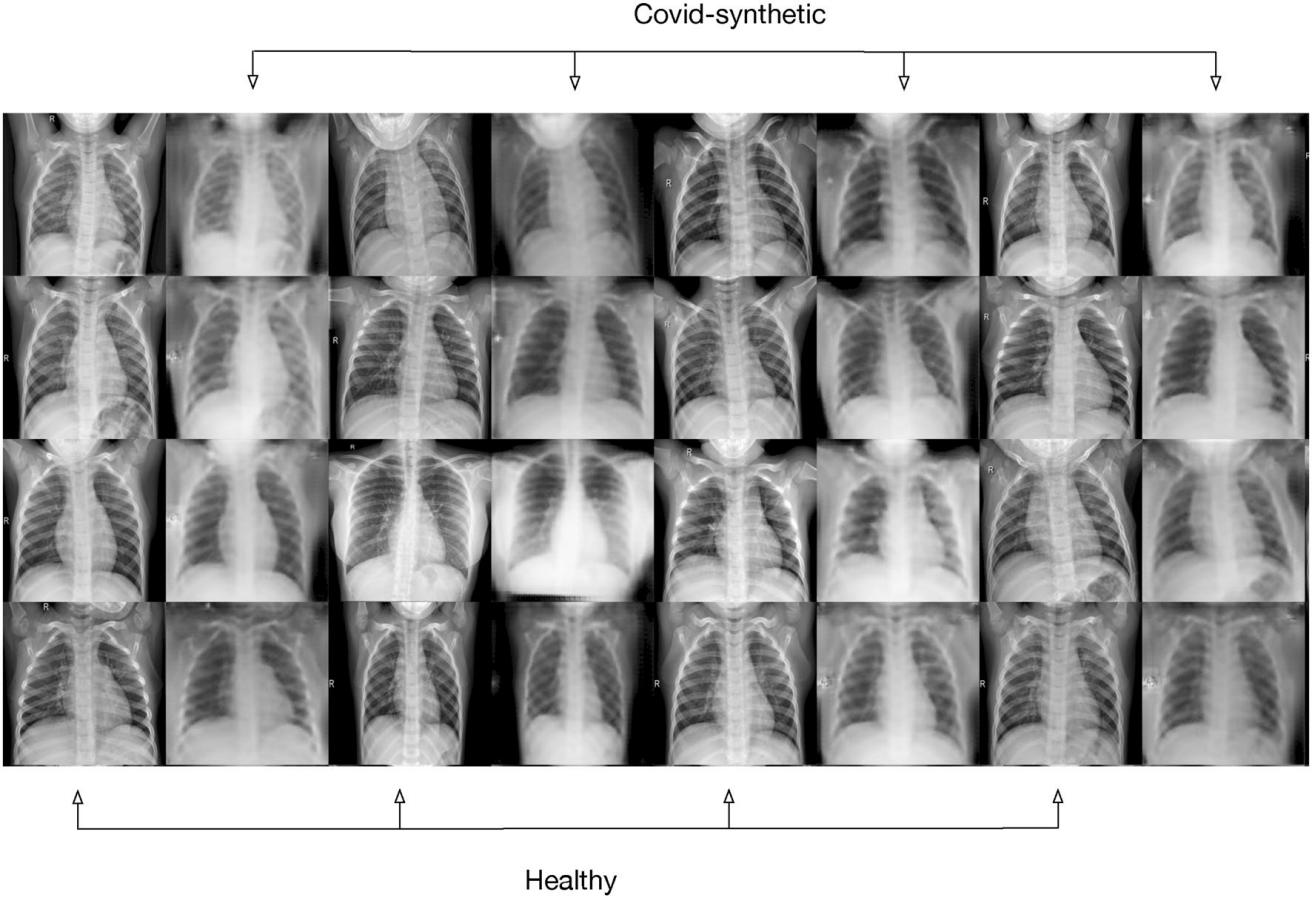
Sample batch of 16 tuples of images after the CycleGAN performed ten thousand iterations. Left: a sample from the *healthy* category, right: a sample from the *covid-synthetic* category

The *covid-synthetic* category was populated with a single batch of 100 synthetic images generated after training for ten thousand epochs (Fig. 6).

The feature space can be observed over the gradient-weighted class activation mapping (Grad-CAM) [23], which uses the gradients flowing into the final convolutional layer to produce a heat map highlighting the features that most contributed to the predicted classification.

**Fig. 7:**
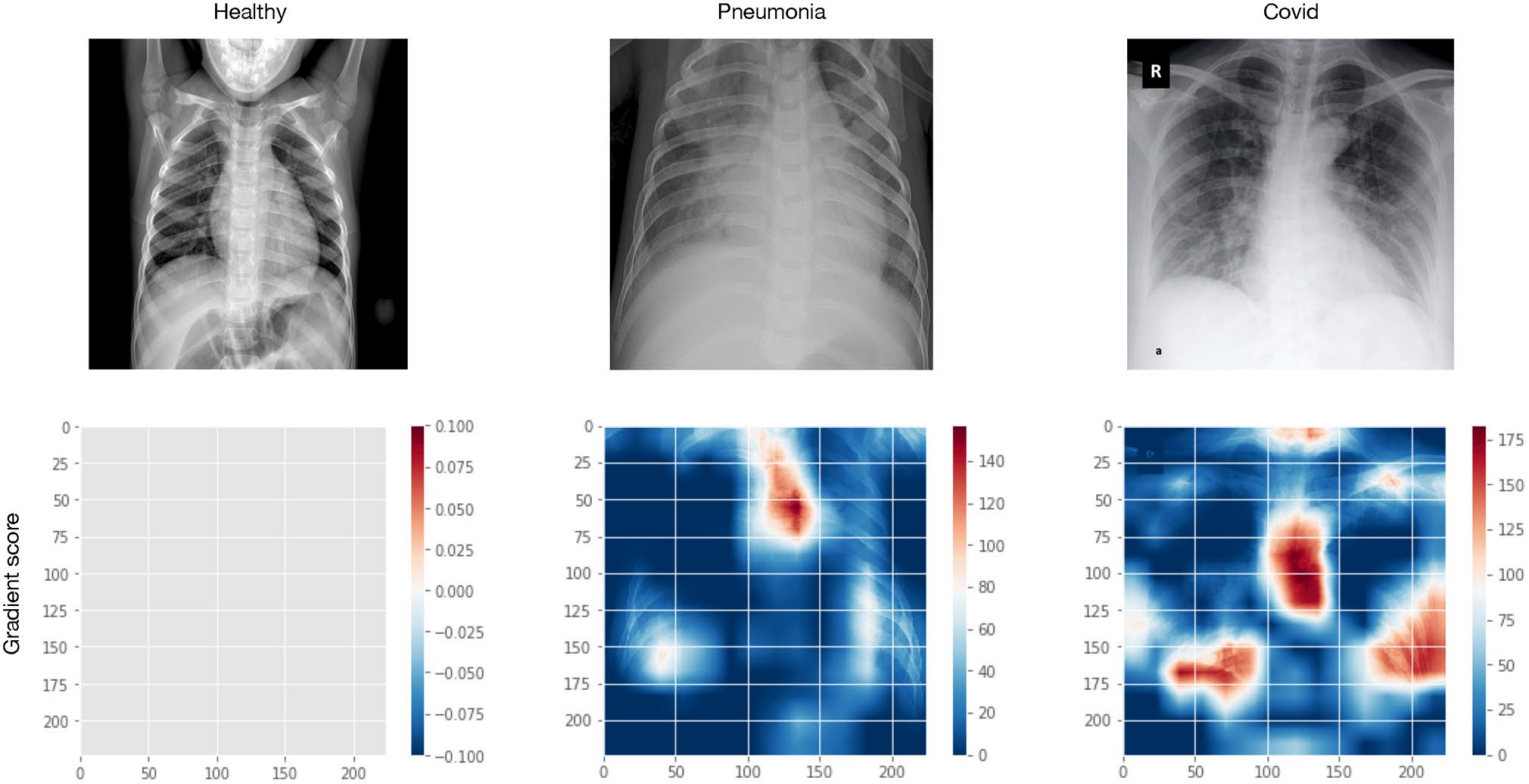
Tuples of X-ray radiographs and their feature space highlighted by Grad-CAM maps. It shows a sample of a healthy individual (left), a patient diagnosed as positive for pneumonia disease (centre), and a patient diagnosed positive for COVID-19 (left)

In Fig. 7, the top images are samples from each of the three categories available in our dataset; below, is a heat map highlighting the feature space characterising each category where the darkest red colour represents the most considerable contribution to classification criteria.

As it can be observed, images from the *covid* category show a higher number of feature areas highlighted in red compared to those from the *pneumonia* category. Also, note that the sample belonging to the *healthy* category does not depict any feature.

**Fig. 8:**
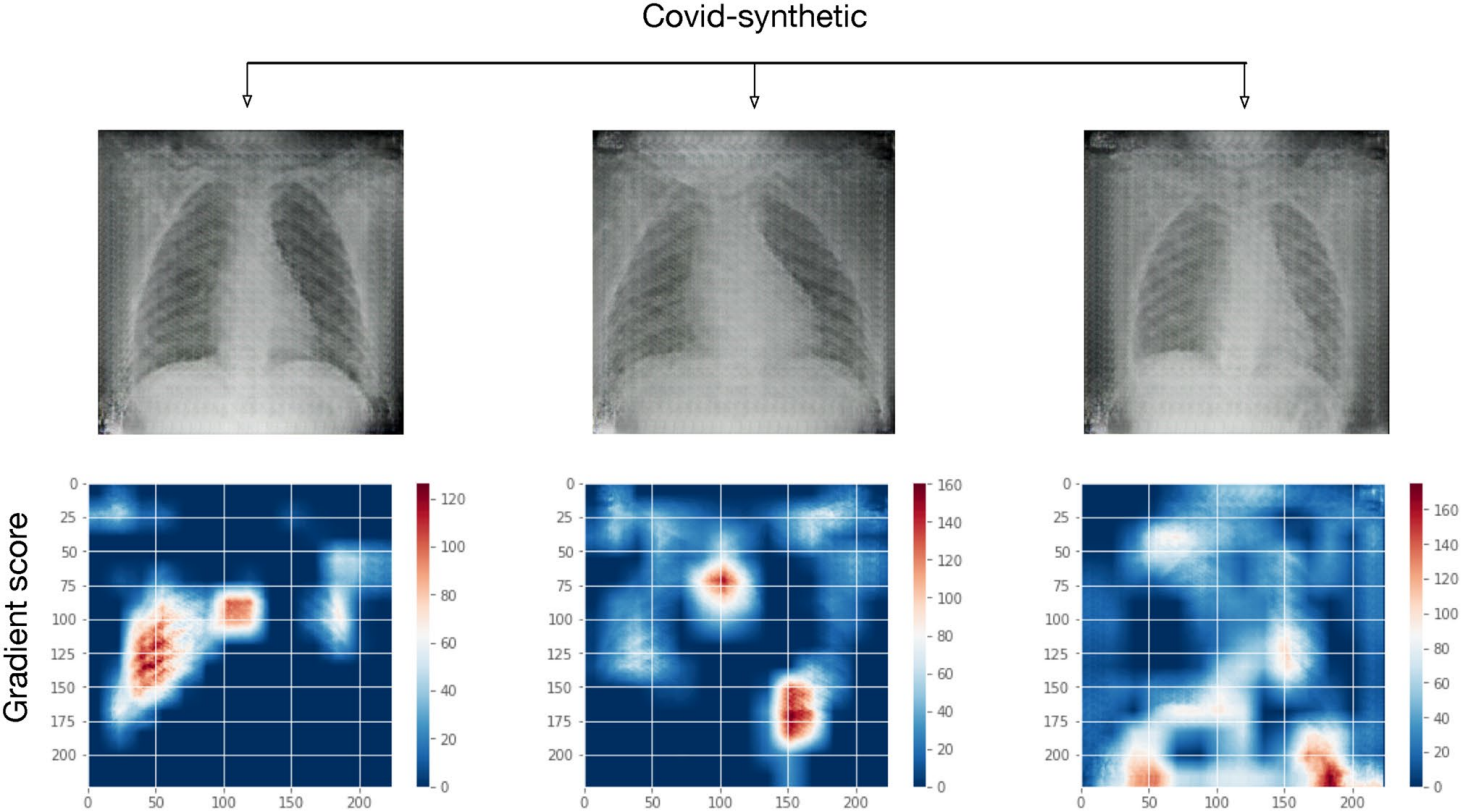
A sample of three synthetically generated images of patients diagnosed positive for COVID-19 taken from category *covid-synthetic*. X-ray radiographs (above) and their feature space highlighted by Grad-CAM maps (below)

In Fig. 8, we present the Grad-CAM mapping over a sample of images from the *covid-synthetic* category. As it can be observed, the feature space captured when using the synthetic images is similar to those observed from the *covid* category.

### Convolutional Neural Network

As this paper is motivated by the insufficient labelled data for training a CNN, we made use of transfer learning techniques (described in Section “Implementation of Transfer Learning Technique for Convolutional Neural Network Architectures”) where we used a small number of images consisting of three categories: *healthy*, *pneumonia*, and *covid* + *covid-synthetic* to fine-tune the last convolution layer of the CNN (see Table 1). We present the results based on the macro f1-score as a metric to evaluate the performance of each CNN model trained over a dataset that gradually integrated the synthetically generated images. As it can be observed in Table 3, the macro *f*1-score gradually increases across the six variations augmented with the synthetic samples. This table shows that the CNN’s performance increases as a larger number of *covid-syntetic* samples are integrated improving their classification accuracy up to 21% in the case of SqueezeNet model when training with datasets augmented by + 90 *covid-synthetic* images.

**Table 3:** Macro *f*1-score that measures the performance of each CNN across six variations in which *covid-synthetic* images are gradually integrated as part of the training of images

CNN	Baseline	+15	+30	+45	+60	+75	+90
AlexNet	0.94	0.94	0.94	0.96	0.94	0.95	0.96
DenseNet	0.77	0.82	0.86	0.83	0.85	0.84	0.87
GoogLeNet	0.94	0.95	0.95	0.94	0.96	0.94	0.95
MNASNet	0.83	0.87	0.86	0.87	0.85	0.88	0.88
ResNet	0.94	0.94	0.95	0.95	0.95	0.96	0.95
ResNeXt	0.93	0.95	0.95	0.95	0.95	0.96	0.96
ShuffleNet	0.92	0.94	0.94	0.95	0.95	0.95	0.95
SqueezeNet	0.65	0.77	0.81	0.83	0.85	0.86	0.86
VGG	0.93	0.94	0.95	0.94	0.95	0.95	0.95
Wide ResNet	0.90	0.92	0.95	0.95	0.94	0.95	0.94

To evaluate the contribution of the CycleGAN-generated images as part of CNN training, we conducted a hypothesis test using a paired sample student’s *t* test statistical analysis. We compared the macro *f*1-score from the baseline against each of the six alternatives that include synthetic images. The calculation was conducted over a one-tailed *t* score.

As it can be observed in Table 4, results across the six CNNs model trained over different variations of data augmentation reject the null hypothesis $H_{0}$ in favour of the alternative hypothesis $H_{1}$ for an $\alpha = 0.05$.

**Table 4:** Statistical calculation of a paired sample *t* test

Alternatives	Mean	Standard deviation	*T* score	*p* value
(+) 15 GAN	0.03	0.04	2.57	0.02
(+) 30 GAN	0.04	0.05	2.64	0.01
(+) 45 GAN	0.04	0.05	2.55	0.02
(+) 60 GAN	0.04	0.06	2.36	0.02
(+) 75 GAN	0.05	0.06	2.57	0.02
(+) 90 GAN	0.05	0.06	2.68	0.01

## Comparison Against Other Methods

Different research has been conducted over chest X-ray radiographs (Section “Implementation of Transfer Learning Technique for Convolutional Neural Network Architectures”). Here we compare our findings with some of the studies that used the same class distribution as we do in this paper (i.e. healthy, pneumonia, COVID-19).

As it can be observed in Table 5, efforts from other researchers attempting different methodologies to model a classifier. These methodologies rely on transfer learning technique with CNNs. A key aspect to observe, however, is the data distribution utilised for training purposes. While their performance is comparable, one of their limitation relying on the imbalance distribution of the datasets adopted for training the models; which one could argue can limit the performance of CNN’s feature modelling.

**Table 5:** Comparison of performance achieved for a multi-class classification problem across different methods trained using chest X-ray images

Study	Data distribution	Methodology	*f*1-score (%)
Tulin et al. [19]	125 COVID-19 500 Pneumonia 500 Healthy	DarkCovidNet	87.02
Arpan et al. [17]	115 COVID-19 3867 Pneumonia 1341 Healthy	CovidAID	92.30
Ioannis et al. [1]	224 COVID-19 700 Pneumonia 504 Healthy	VGG-19	93.06
Sethy et al. [23]	127 COVID-19 127 Pneumonia 127 Healthy	ResNet50 + SVM	95.52

When comparing the related work against the higher CNN modelled with covid-synthetic (AlexNet, ResNeXt), one can observe that the classification performance is similar. Nonetheless, our findings suggest that the performance of a CNN can be unaffected in scenarios where there are as little as 15 labelled samples, as one can rely on synthetic samples to balance a dataset.

## Discussion and Conclusion

The contribution of this work was to illustrate the impact of neural style transfer as an approach for alleviating the insufficiency of labelled samples in imbalanced datasets when modelling image classification problems. Mainly, we focused on the task of chest X-ray radiograph for the diagnosis of COVID-19 as a computational alternative to accelerate the development of machine learning technology to assist in the diagnosis of this medical condition.

As the neural style transfer refers to techniques enabled to adopt key characteristics from one set of samples to another to perform image transformation, in this paper, we used a CycleGAN to generate a set of samples from X-ray images of healthy individuals (widely available) so that they look as if they belong to an X-ray image set of patients diagnosed positive for COVID-19 (*covid-synthetic*), scarcely available. To evaluate this approach, we made use of transfer learning techniques by retraining some of the most successful CNNs. The evaluation consisted of assessing the macro *f*1-score across six different alternatives in which *covid-synthetic* images are gradually incorporated as part of the training set of images.

It can be observed that the classification accuracy from the baseline is generally high across the different CNNs considering the small number of samples used at the training stage (*n* = 15), which we attribute to the adoption of the transfer learning technique over the balanced dataset used.

As presented in Table 5, our findings suggest that by adopting synthetic images, training CNNs with transfer learning techniques preserves the performance of feature mining, which can be an important contribution in scenarios of emerging phenomena (such as COVID-19), and more generally in situations of limited data availability.

The ability to extract features relevant to an image category is determined by diverse factors such as the convolutional design of CNN layers or the number of training samples within a category. In this work, one can observe that as the synthetic images are gradually incorporated as part of the training set, the classification accuracy increases across the different CNN architectures, which we have shown is likely due to the contribution of *covid-synthetic* images used as data augmentation to training the different CNN models.

In summary, our results show the feasibility of using synthetic X-ray images to alleviate the insufficiency of a radiograph of patients diagnosed positive for COVID-19. While the statistical results show confidence for a one-tailed *t* score = 2.68 and *p* value = 0.01 for $\alpha =0.05$, we encourage the community to experiment further with the neural style transfer approach to generating synthetic images. Building upon innovative style transfer techniques can accelerate the development of image classification algorithms to cope with the insufficiency of labelled data in erratic scenarios of emerging clinical contingencies.

## Footnotes

1 https://www.nvidia.com/en-us/data-center/v100/.
